# China Elite Athletes Cardiovascular hEath (China‐ACE) Study: A Protocol for a Multicenter Prospective Cohort

**DOI:** 10.1002/clc.70241

**Published:** 2025-12-23

**Authors:** Sheng Xu, Yaodong Guo, Hao Xin, Shanshan Zhuo, Yang Zhao, Yanna Jiang, Yuanqi Huang, Chunyan Huang, Tao Chen, Qi Chen, Qianru Zhao, Dan Wang, Xiao Liu, Zhiwei Yan

**Affiliations:** ^1^ Department of Ultrasound Medicine Jinqiu Hospital Shenyang Liaoning China; ^2^ School of Physical Education and Sport Sciences Fujian Normal University Fuzhou Fujian China; ^3^ Department of Ultrasound The First Affiliated Hospital of Fujian Medical University Fuzhou Fujian China; ^4^ Liaoning Provincial Sports Development Center Shenyang Liaoning China; ^5^ Shenyang Sports Research and Medical Center, Shenyang Sports Development Center Shenyang Liaoning China; ^6^ Department of Cardiology Sun Yat‐sen Memorial Hospital of Sun Yat‐sen University Guangzhou China; ^7^ Cardiovascular and Metabolic Disorders Program, Duke‐National University of Singapore Medical School Singapore Singapore

**Keywords:** athletes, cardiovascular function, dose‐response relationship, sex, sports

## Abstract

**Background and Objective:**

Exercise mediates cardiac adaptation in a dose‐dependent manner, and each sport has unique demands on the cardiovascular system. Given the long‐term effects of key confounding variables such as age, sex, and exercise dose, knowledge gained from one sport cannot be generalized to all sports. Therefore, it is necessary to explore the potential dose‐response relationships between different sports and cardiovascular adaptations on a broader time scale, and to explore the regulatory role of key factors such as age and sex.

**Methods:**

The China‐ACE study will be a prospective longitudinal cohort study involving approximately 500 elite athletes of different sexes, ages, and sports (rowing, canoeing, cycling, triathlon, weightlifting, wrestling, judo, swimming, basketball, badminton, boxing, and beach volleyball) from three provincial sports centers. All athletes will undergo two screenings within 1 year, during which their exercise load and dose will be recorded via quantitative and semi‐quantitative methods. The screening will include measuring cardiovascular function, body composition, electrocardiogram, heart rate variability, echocardiography, cardiopulmonary function, circulating biochemical markers, sex hormones, lower limb muscle function, and physical performance.

**Conclusion:**

The China‐ACE study will elucidate the potential dose‐response relationship between exercise load and cardiovascular adaptation, cardiopulmonary function, skeletal muscle function, and physical performance by continuously tracking the exercise load and exercise dose of athletes in different sports and exploring the potential role of age and sex in these adaptations. This may inform individualized training and cardiovascular prevention for athletes and extend the current understanding of sports cardiology.

## Graphical abstract

This year‐long, large‐scale cohort study will examine how cardiovascular adaptations vary across different sports disciplines, exploring the dose‐response relationship and assessing the influence of age, sex, and years of training.

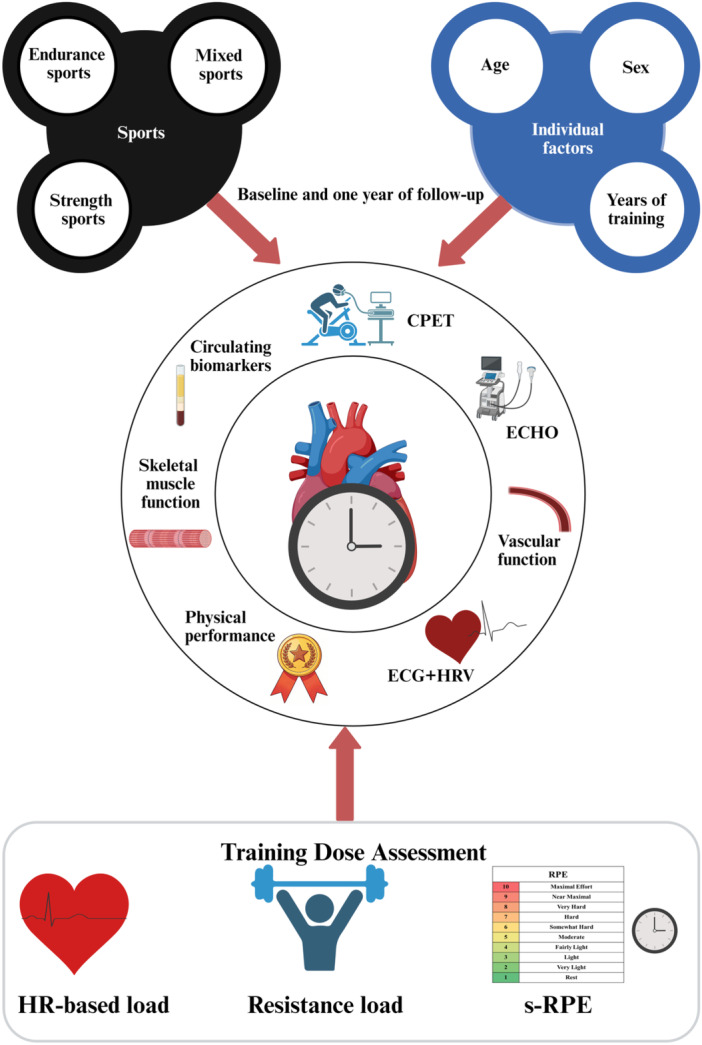

**Abbreviations**
ECG: electrocardiogram
ECHO: echocardiogram
HRV: heart rate variability
LVWT: left ventricular wall thickness

## 1 Introduction

Regular exercise induces structural and functional adaptations in the heart, which are closely related to the exercise dose and are often dose‐dependent [1]. The hearts of competitive athletes undergo a wide range of structural, functional, and electrophysiological adaptations in response to significantly increased hemodynamic loads during prolonged strenuous exercise [2, 3]. Exercise‐induced cardiac adaptations are heterogeneous and can vary significantly by sport, sex, and age [4]. Sports focus on exercise‐induced cardiac adaptation and a key factor contributing to differences in exercise‐induced cardiac adaptation. Currently, most of the evidence on differences in cardiac adaptation due to sports is based on cross‐sectional data [5]. Strength, endurance, and mixed sports lead to different cardiac adaptations due to hemodynamic factors such as changes in pressure and volume [6]. Endurance sports lead to enlargement of both the left and right ventricles with bradycardia, increased right ventricular diameter, increased end‐diastolic volume, and enhanced diastolic function, whereas left ventricular wall thickness (LVWT) remains unchanged or increases slightly [5, 7, 8]. Athletes who perform strength sports typically experience an increase in LVWT and small changes in left ventricular size [7]. Importantly, each sport has unique demands on the cardiovascular system and insights gained from one sport may not apply to others. Therefore, exploring the characteristics of exercise‐mediated cardiac adaptations across a wide range of sports is necessary. There is also a lack of longitudinal cohort data on different sports versus exercise doses, which may shed light on some of the current disputes in this area.

Sex also plays a key role in the regulation of exercise‐induced heart. Compared with male athletes, female athletes typically lack significant and abnormal electrophysiologic changes [9]. In addition, there are significant sex differences in cardiac structural adaptations, with male athletes typically exhibiting greater LVWT and significant end‐diastolic volume changes [10]. In endurance exercise‐induced morphological adaptations of the heart, female athletes are more likely to show centrifugal remodeling, whereas some male athletes may show centripetal remodeling [11]. Notably, the lower cardiac response to exercise in females than in males is not simply due to the lower participation of female athletes in sports or the lower exercise dose (exercise intensity and duration), but may be related to the unique neuroendocrine regulation in females [12, 13].

Cardiac adaptations induced by prolonged exercise training begin to appear during adolescence and progressively develop with age [14]. Within the same sports, adolescent males generally exhibit significantly lower left and right ventricular mass and volume compared to adult male athletes [5]. However, there is no significant sex difference in the left and right ventricular volumes or masses in females [15]. Notably, cardiac adaptations in adolescent skiers initially present as concentric remodeling, which gradually shifts to eccentric remodeling with age, and may revert to concentric remodeling in adulthood [14]. However, most existing studies have focused on adult athletes and lack data comparing cardiovascular adaptation in adolescent athletes from different sports. In addition, there is also a lack of longitudinal data looking at cardiac adaptation in adolescents, as this can provide us with information on the effects of exercise dose and timing.

Notably, each sport has unique demands on the cardiovascular system, and lessons learned from one sport may not be applicable to another, given the long‐term effects of key confounding variables such as age, sex, and exercise dose (intensity and duration). Therefore, there is a need to explore potential dose‐response relationships between different sports and cardiac adaptations from a broader temporal spectrum and explore the regulatory role of key factors such as age and sex. This could inform individualized training and cardiovascular prevention in athletes and extend current understanding of sports cardiology.

## 2 Materials and Methods

### 2.1 Research Design

The program is a multicenter prospective longitudinal cohort study in which all athletes will be screened twice initially and 1 year later to collect cardiovascular health data, circulating biochemical markers, sex hormones, skeletal muscle function, and physical performance. The Ethics Committee of Fujian Normal University approved the study protocol (Approval Code: 2024‐7‐10). All athletes will be provided written informed consent before enrollment, and the study results will be reported following the Strengthening the Reporting of Observational Studies in Epidemiology (STROBE) guidelines [16].

### 2.2 Research Setting

Based on the reserve of athletes in the Liaoning Provincial Sports Center, Zhejiang Provincial Sports Center, and Fujian Provincial Sports Center, we will recruit 500 eligible elite athletes to participate in this study. The informational session will be held for athletes and coaches to explain the project content and setup. Athletes are categorized based on European Society of Cardiology (ESC) criteria into endurance sports (swimming, triathlon, cycling, rowing, and canoeing), strength sports (weightlifting, judo, wrestling, and boxing), and mixed sports (badminton, basketball, and beach volleyball) [17]. In addition, Athletes are categorized by age into young athletes (≤ 18 years), adult athletes (19–34 years), and master athletes (≥ 35 years) [18]. Athletes were initially enrolled in June 2024 and the recruitment is still ongoing. Initial recruitment is expected to be completed by December 2025 due to the dispersed nature of the study centers and the difficulty in recruiting athletes. Data collection is expected to be completed by June 2027, after approximately 1 year of follow‐up. The results of the study will be analyzed and presented after all data collection has been completed.

### 2.3 Inclusion Criteria

Participants will be included for both of the following criteria: (1) Athletes who meet the definition of elite‐level athletes (participants must have ranked in the top six at the National Senior Championships or the top three at the National Junior Championships); (2) Athletes from the designated regions specified in the study; (3) Athletes who can consistently participate in training and remain in the study for the next year; (4) No use of hormonal contraceptives within the 3 months before enrollment [19].

### 2.4 Exclusion Criteria

Participants who meet the following criteria will be excluded: (1) Athletes with a history of diagnosed cardiovascular disease; (2) A first‐degree relative with a history of severe cardiovascular disease (e.g., heart failure, hypertrophic cardiomyopathy, hereditary arrhythmia syndrome) or who was diagnosed with definite major cardiovascular disease symptoms.

Notably, for athletes with non‐immediate family members who have severe cardiovascular diseases, we will arrange regular electrocardiogram (ECG) and echocardiogram (ECHO) examinations to increase safety monitoring.

### 2.5 Measurements

Research assistants will collect baseline data from participants using registration forms, which will capture age, sex, sports, and years of training. The primary outcomes will assess cardiac structure and systolic/diastolic function through ECHO. Secondary outcomes will include evaluations of body composition, cardiovascular function, ECG, heart rate variability (HRV), cardiopulmonary function, circulating biochemical markers, sex hormone levels, lower limb muscle function, and overall physical performance. All laboratory assessments will be conducted on the first training day following a rest day, both at baseline and at the 1‐year follow‐up. The detailed measurement parameters and the instruments used for each assessment are provided in Table 1. Participants will be instructed to avoid strenuous physical activity and to abstain from alcohol, caffeine, or any substances that may influence cardiovascular function within 24 h before testing. For female participants, menstrual cycle phases will be verified via both self‐report questionnaires and specialized menstrual test strips. Training loads will be continuously tracked throughout the 1 year using athlete‐maintained training logs or diaries.

**Table 1:** Overview of measurement methods and evaluation tools.

Outcome measures	Indicator	Instrument
Body composition	Total fat mass, total muscle mass, body fat percentage	Body composition analyzer (InBody 770, InBody Co, Korea)
Blood pressure	Systolic blood pressure, diastolic blood pressure, pulse pressure, rate pressure product	Automated sphygmomanometer (Omron HEM 3, Omron, Japan)
Arterial stiffness	Brachial‐ankle pulse wave velocity	Vascular function analyzer (BP‐203RPEⅢ, Omron, Japan)
Cardiac electrophysiological function	HR, P(ms), PR(ms), QRS(ms), QTc(ms), P/QRS/T(deg), RV5/SV1(mV), RV5 + SV1(mV), RV6/SV2(mV)	12‐lead ECG (EDAN SE‐1010, China)
Autonomic nervous system	Time‐domain: SDNN, RMSSD, RR_max_, RR_mean_, RR_min_, Tempo (s), Misure, NN50, PNN50, and SMisure, Frequency‐domain: VLF, LF, HF, VLF%, LF%, HF%, LFnu, HFnu, LF/HF, LFnu/HFnu, ln(LF), ln(HF), ln(LF)/ln(HF). Nonlinear: SD1, SD2, SD1/SD2	HRV (EDAN SE‐1010, China)
Cardiac structure	IVST, LVPWT, LVEDD, LVESD, RV1, RV2, LVEDV, LVESV, RWT, LVM, LVMI	GE VIVID E95 ultrasound system equipped (GE Vingmed Ultrasound, Horten, Norway)
Atrial structure	LAD, RA major, RA minor	GE VIVID E95 ultrasound system equipped (GE Vingmed Ultrasound, Horten, Norway)
Vascular structure	Ao root, Asc Ao, PA	GE VIVID E95 ultrasound system equipped (GE Vingmed Ultrasound, Horten, Norway)
Myocardial diastolic function	LVEF, TAPSE, AV Vmax, PV Vmax	GE VIVID E95 ultrasound system equipped (GE Vingmed Ultrasound, Horten, Norway)
Myocardial systolic function	E, A, E/A, e′, a′, e′_avg_, E/e'	GE VIVID E95 ultrasound system equipped (GE Vingmed Ultrasound, Horten, Norway)
Complete blood count	white blood cell count, red blood cell count, hemoglobin concentration, hematocrit, platelet count, lymphocyte count, monocyte count, granulocyte count	Fully automated hematology analyzer (MEK‐6410P, Nihon Kohden, Japan)
Enzymes	Aspartate aminotransferase, lactate dehydrogenase, amylase, lipase, CK, CK‐MB, α‐hydroxybutyrate dehydrogenase, high‐density lipoprotein, low‐density lipoprotein, triglycerides, total cholesterol	Fully automated biochemical analyzer (SD1, Seamaty, China)
Electrolytes	Mg, Cl^−^, Na^+^, K^+^	Fully automated biochemical analyzer (SD1, Seamaty, China)
Trace elements	Calcium, phosphorus	Fully automated biochemical analyzer (SD1, Seamaty, China)
Metabolites	Creatinine, uric acid, urea, urea/creatinine, glucose, total CO2	Fully automated biochemical analyzer (SD1, Seamaty, China)
Inflammatory markers	c‐reactive protein	Fully automated biochemical analyzer (SD1, Seamaty, China)
Sex hormones	Luteinizing hormone, follicle‐stimulating hormone, sex hormone‐binding globulin, estradiol, progesterone, testosterone	ELISA analyzer (AMR‐100, Allsheng, China)
Cardiopulmonary function	AT, VO_2max_	VO2000 (MedGraphics Corp, USA)
Skeletal muscle function	Muscle tone, elasticity, and stiffness	Myoton PRO (Myoton AS, Tallinn, Estonia)

Abbreviations: a′, late diastolic mitral annular velocity; A, peak A wave velocity; Ao root, Aortic root diameter; Asc Ao, Ascending aorta diameter; AV Vmax, Aortic valve flow peak velocity; CK, creatine kinase; CK‐MB, creatine kinase‐MB; e′, The early diastolic mitral annular velocity; E, Peak E wave velocity; e′avg, the average velocity of the mitral annulus; IVST, Interventricular septum thickness; LAD, Left atrial diameter; LVEDD, left ventricular end‐diastolic diameter; LVEF, left ventricular ejection fraction; LVEDV, left ventricular end‐diastolic volume; LVESD, left ventricular end‐systolic diameter; LVESV, left ventricular end‐systolic volume; LVM, left ventricular mass; LVMI, left ventricular mass index; LVPWT, left ventricular posterior wall thickness; PA, Pulmonary artery diameter; PV Vmax, Pulmonary artery valve flow peak velocity; RA major, Right atrium superior‐inferior diameter; RA minor, Right atrium transverse diameter; RVD1, RV basal diameter; RVD2, RV mid‐cavity diameter; RWT, relative wall thickness; TAPSE, Tricuspid annular plane systolic excursion.

### 2.6 Cardiovascular Assessment and Follow‐Up

All athletes will undergo a standardized cardiovascular screening protocol consisting of a physical examination, ECG, and ECHO. The physical examination will include general health assessments such as blood pressure measurement, height, weight, and body composition analysis. ECG and ECHO results will be interpreted in accordance with current cardiovascular screening guidelines for athletes [20, 21]. Any athlete presenting with abnormal findings in blood pressure, ECG, or ECHO parameters during screening will be documented and placed under close clinical observation. If an athlete with such abnormalities experiences adverse cardiovascular symptoms during training or testing—such as palpitations, chest pain, pronounced chest tightness, dizziness, or presyncope—exercise will be immediately halted. The coaching and medical teams will be notified promptly, and specialist evaluation and follow‐up will be arranged as necessary.

### 2.7 Data Management

All electronic data will be centrally stored and managed by an independent researcher who is not involved in the study design or implementation. Any data extraction will require approval from all investigators. To safeguard participant privacy, each athlete will be assigned a unique, permanent identification code. This code will replace personal identifiers in all data collection, analysis, and reporting throughout the study and follow‐up periods. All study‐related records will be securely retained for a minimum of 15 years following the completion of follow‐up for the last enrolled athlete.

### 2.8 Statistical Analysis

Baseline characteristics will be summarized using descriptive statistics. Continuous variables will be presented as means ± standard deviations or medians with interquartile ranges, depending on their distribution, while categorical variables will be reported as frequencies and percentages. For group comparisons, analysis of variance (ANOVA) or the Kruskal–Wallis test will be used for continuous variables, and the *χ* ^2^ test will be applied for categorical variables.

A sample size of 500 elite athletes was determined primarily by feasibility and resource availability based on the athlete reserve in the participating centers. The primary objective is to investigate the impact of training load on the change of cardiac structural and functional in elite athletes. According to a previous study, this sample size of 500 will provide robust statistical power (> 90%) to detect clinically relevant cardiac structural and functional (e.g., interventricular septum thickness, left ventricular end‐diastolic volume) changes over 1 year of follow‐up [22]. Secondary outcomes will be changes in cardiopulmonary function, circulating biochemical markers, sex hormone levels, lower‐limb muscle function, and physical performance, blood pressure, body composition evaluation, ECG, and HRV.

To assess the association between exercise dose and changes in cardiovascular outcomes, linear mixed‐effects (LMMs) models will be employed. The models will include fixed effects for time (baseline vs. 1‐year), sport category (endurance, strength, mixed), age (adolescent vs. adult), years of training, sex (male, female), study center and exercise dose. Random effects will be included for each participant to account for between‐subject variability at baseline and specific sport category (e.g., rowing, cycling). Interaction terms for sport category, sex, years of training and exercise dose will be included. A sensitivity analysis by using multiple imputation will be applied. To examine the potential non‐linear dose‐response relationship between exercise load and continuous cardiovascular outcomes, we employed restricted cubic splines within the linear mixed‐effects model. All statistical analyses will be conducted using the R program (version 4.4.1). A two‐sided *p* **< **0.05 will be considered statistically significant.

## 3 Discussion

This study will be the first large‐scale longitudinal cohort study in Asia to systematically assess cardiovascular health, sex hormones, skeletal muscle function, physical performance, and exercise dosage in elite athletes. Approximately 500 outstanding athletes of different ages and sexes from strength, endurance, and mixed sports will participate. Consistent with previous large longitudinal studies in athletes conducted in Europe and the United States, this protocol will use electrophysiological and imaging assessments to evaluate sport‐specific adaptations in cardiac structure and function across different sports [23, 24, 25]. However, the relationship between exercise‐induced cardiovascular adaptations and exercise dose remains poorly defined. Therefore, this study will take a combination of quantitative and semiquantitative methods to monitor the exercise dose of athletes over the course of a year. The study will thoroughly investigate the dose‐response relationship, as well as its associations with different sports, age, and sex.

The athlete's heart requires repeated and sustained exposure to a certain dose of exercise stimuli. Exercise dose can be characterized by exercise intensity and duration, which vary significantly from program to program due to differences in mobilized muscle types and metabolic patterns. Currently, the minimum exercise dose required is still largely out of consensus. This knowledge gap is due to the fact that most of the available evidence is based on cross‐sectional studies, which provide information on the structural and functional characteristics of the heart in a given program and do not provide insights into the dose‐response relationship and temporal effects between sports and cardiac adaptations [5]. Insights into the dose‐response and temporal relationships between different programs and cardiac adaptations need to be explored from broader temporal spectrum analyses. Longitudinal studies provide more information on exercise dose and time‐action, but continuous quantification of exercise dose remains a challenge. As a result, there are only a few longitudinal studies on exercise‐based cardiac adaptations, but they suffer from the drawbacks of short exposure times, small sample sizes, and single programs [26, 27, 28]. Notably, each sport has unique demands on the cardiovascular system, and lessons learned from one sport may not be applicable to another. Sex plays a key role in exercise‐induced cardiac adaptation. To accurately portray the dose‐response relationship of exercise, this study will use quantitative and semi‐quantitative methods to collect exercise loads, including those coming from athletes in strength, endurance, and mixed events, and explore the role of exercise programs in exercise cardiac adaptation. Sex plays a key role in exercise‐induced cardiac adaptation. Currently, most of the knowledge on sex differences in exercise‐related cardiac adaptations is derived from cross‐sectional studies [29]. Compared with male athletes, female athletes generally lack abnormal ECG changes and significant cardiac remodeling, and have good blood pressure regulation ability during exercise, which is thought to be related to the unique cardiovascular protection mechanism in women [30]. Sex hormones and cardiac autonomic nerves are thought to play important roles in this specific protection. Sex hormones play a completely different role in the exercise‐induced heart. Estrogen has a protective effect on cardiomyocytes and inhibits cardiac hypertrophy by suppressing the expression of proteins such as calmodulin phosphatase [12, 31]. Instead, testosterone acts directly on specific receptors in cardiomyocytes, promoting cardiac hypertrophy by promoting amino acid and protein synthesis [13]. In addition, autonomic tone during exercise shows significant sex differences, with significantly increased sympathetic tone in males and greater parasympathetic dominance in females, which may lead to significant hemodynamic and cardiovascular response differences [32, 33]. Although cross‐sectional studies revealing sex differences have increased in recent years, there is relatively little longitudinal evidence on cardiac adaptation in female athletes. Therefore, this study evaluated potential sex differences in cardiovascular structure, function, and electrophysiological adaptation of endurance, strength, and mixed exercise over a 1‐year period through a longitudinal study. Given the role of sex hormones and cardiac autonomic nerves in sex differences in exercise‐related cardiac adaptation, evidence regarding changes in sex hormones and cardiac autonomic function remains insufficient in large longitudinal studies of athletes in Europe and the United States. This study will also comprehensively examine the levels of sex hormones, such as estradiol, luteinizing hormone, follicle‐stimulating hormone, progesterone, prolactin, testosterone, and analyze sympathetic‐vagal tone by linear and nonlinear methods, which could serve as mechanisms to explain the characterization of sex differences in exercise cardiac.

The hearts of elite athletes exhibit greater plasticity during adolescence, and as they transition into adulthood, their hearts gradually mature and undergo exercise‐induced adaptive remodeling [34]. However, most existing studies have focused on adult athletes and lack data comparing cardiovascular adaptation in adolescent athletes from different sports. In addition, there is also a lack of longitudinal data looking at cardiac adaptation in adolescents, as this can provide us with information on the effects of exercise dose and timing. Therefore, this study will use a longitudinal study to compare differences in cardiovascular adaptation between adolescent and adult elite athletes in the same sport and explore the potential role of exercise dose in adolescent cardiac adaptation.

Finally, the lower limb muscle function and physical performance of the athletes will be evaluated. As an integrated index reflecting the cardiovascular function, cardiopulmonary function, and skeletal muscle function of athletes, sports performance can give us a more comprehensive insight into the effects of exercise dose and time on the exercise‐induced heart. Therefore, a long‐term longitudinal study that integrates key confounding variables such as sport, age, sex, exercise dose, and time will expand the current understanding of the exercise heart.

### 3.1 Limitation

This study has several limitations. First, due to constraints in experimental conditions, the included sports were limited to endurance, strength, and mixed modalities; skill‐based sports were excluded. As a result, the study does not fully capture the spectrum of cardiac adaptations associated with different athletic sports. Second, the participant pool was limited to individuals of Asian descent, reflecting geographic and demographic constraints. Consequently, the findings may not be generalizable to athletes from other ethnic backgrounds.

## 4 Conclusion

The China‐ACE study will explain the potential dose‐response relationships between exercise load and cardiovascular adaptation, cardiopulmonary function, skeletal muscle function, and exercise performance by continuously tracking exercise load and exercise dose of athletes in different sports, and exploring the potential role of age and sex in these adaptations. This may inform individualized training and cardiovascular prevention for athletes and expand the current understanding of sports cardiology.

## Author Contributions

Zhiwei Yan was responsible for the study design; Sheng Xu, Yaodong Guo, and Zhiwei Yan participated in drafting the initial version of the manuscript, while Zhiwei Yan, Xiao Liu, and Sheng Xu further contributed to data interpretation and manuscript revisions. Yaodong Guo, Xiao Liu, and Zhiwei Yan prepared the final version of the manuscript.

## Ethics Statement

The Ethics Committee of Fujian Normal University approved the study protocol (Approval Code: 2024‐7‐10). All athletes will provide written informed consent before enrollment.

## Consent

All athletes will provide written informed consent before enrollment.

## Conflicts of Interest

The authors declare no conflicts of interest.

## Supporting information

ptorocol supplemtary material.

## Data Availability

The authors have nothing to report.

## References

[1] B. A. Franklin , P. D. Thompson , S. S. Al‐Zaiti , et al., “Exercise‐Related Acute Cardiovascular Events and Potential Deleterious Adaptations Following Long‐Term Exercise Training: Placing the Risks Into Perspective‐An Update: A Scientific Statement From the American Heart Association,” Circulation 141, no. 13 (2020): e705–e736. 32100573 10.1161/CIR.0000000000000749

[2] D. Niederseer , V. A. Rossi , C. Kissel , et al., “Role of Echocardiography in Screening and Evaluation of Athletes,” Heart 107, no. 4 (2021): 270–276. 10.1136/heartjnl-2020-317996 33203709

[3] D. Corrado , A. Biffi , C. Basso , A. Pelliccia , and G. Thiene , “12‐lead ECG in the Athlete: Physiological Versus Pathological Abnormalities,” British Journal of Sports Medicine 43, no. 9 (2009): 669–676. 19734501 10.1136/bjsm.2008.054759

[4] M. W. Martinez , J. H. Kim , A. B. Shah , et al., “Exercise‐Induced Cardiovascular Adaptations and Approach to Exercise and Cardiovascular Disease,” Journal of the American College of Cardiology 78, no. 14 (2021): 1453–1470. 34593128 10.1016/j.jacc.2021.08.003

[5] P. Spirito , A. Pelliccia , M. A. Proschan , et al., “Morphology of the “Athlete's Heart” Assessed by Echocardiography in 947 Elite Athletes Representing 27 Sports,” American Journal of Cardiology 74, no. 8 (1994): 802–806. 7942554 10.1016/0002-9149(94)90439-1

[6] R. Kovacs and A. L. Baggish , “Cardiovascular Adaptation in Athletes,” Trends in Cardiovascular Medicine 26, no. 1 (2016): 46–52. 25976477 10.1016/j.tcm.2015.04.003

[7] J. Morganroth , B. J. Maron , W. L. Henry , and S. E. Epstein , “Comparative Left Ventricular Dimensions in Trained Athletes,” Annals of Internal Medicine 82, no. 4 (1975): 521–524. 1119766 10.7326/0003-4819-82-4-521

[8] A. D'Andrea , L. Riegler , S. Morra , et al., “Right Ventricular Morphology and Function in Top‐Level Athletes: A Three‐Dimensional Echocardiographic Study,” Journal of the American Society of Echocardiography 25, no. 12 (2012): 1268–1276. 22898244 10.1016/j.echo.2012.07.020

[9] B. J. Petek , E. H. Chung , J. H. Kim , et al., “Impact of Sex on Cardiovascular Adaptations to Exercise,” Journal of the American College of Cardiology 82, no. 10 (2023): 1030–1038. 37648352 10.1016/j.jacc.2023.05.070

[10] S. V. Wooten , S. Moestl , P. Chilibeck , et al., “Age‐ and Sex‐Differences in Cardiac Characteristics Determined by Echocardiography in Masters Athletes,” Frontiers in Physiology 11 (2021): 630148. 33536945 10.3389/fphys.2020.630148 PMC7848176

[11] G. Finocchiaro , H. Dhutia , A. D'Silva , et al., “Effect of Sex and Sporting Discipline on LV Adaptation to Exercise,” JACC: Cardiovascular Imaging 10, no. 9 (2017): 965–972. 27865722 10.1016/j.jcmg.2016.08.011

[12] T. Luo and J. K. Kim , “The Role of Estrogen and Estrogen Receptors on Cardiomyocytes: An Overview,” Canadian Journal of Cardiology 32, no. 8 (2016): 1017–1025. 26860777 10.1016/j.cjca.2015.10.021 PMC4853290

[13] J. L. Vingren , W. J. Kraemer , N. A. Ratamess , J. M. Anderson , J. S. Volek , and C. M. Maresh , “Testosterone Physiology in Resistance Exercise and Training: The Up‐Stream Regulatory Elements,” Sports Medicine 40, no. 12 (2010): 1037–1053. 21058750 10.2165/11536910-000000000-00000

[14] A. W. Bjerring , H. E. W. Landgraff , S. Leirstein , et al., “From Talented Child to Elite Athlete: The Development of Cardiac Morphology and Function in a Cohort of Endurance Athletes From Age 12 to 18,” European Journal of Preventive Cardiology 28, no. 10 (2021): 1061–1067. 33611558 10.1177/2047487320921317

[15] I. Csecs , C. Czimbalmos , A. Toth , et al., “The Impact of Sex, Age and Training on Biventricular Cardiac Adaptation in Healthy Adult and Adolescent Athletes: Cardiac Magnetic Resonance Imaging Study,” European Journal of Preventive Cardiology 27, no. 5 (2020): 540–549. 31370686 10.1177/2047487319866019

[16] J. P. Vandenbroucke , E. von Elm , D. G. Altman , et al., “Strengthening the Reporting of Observational Studies in Epidemiology (STROBE): Explanation and Elaboration,” Annals of Internal Medicine 147, no. 8 (2007): W163‐94. 17938389 10.7326/0003-4819-147-8-200710160-00010-w1

[17] S. Sharma , S. Gati , M. Bäck , et al., “2020 ESC Guidelines on Sports Cardiology and Exercise in Patients With Cardiovascular Disease,” European Heart Journal 42 (2021): 17À96. 33180902 10.1093/eurheartj/ehaa735

[18] G. Gaggioli , M. Brignole , D. Poggi , S. Iacovelli , L. Zuccarino , and S. Castelletti , “Pre‐Participation Screening in Competitive Athletes in Italy: An Analysis of Real‐World Practice From a Large Database,” European Journal of Preventive Cardiology, ahead of print, July 29, 2025, 10.1093/eurjpc/zwaf454. 40729124

[19] A. Lafitte , M. Dupuit , T. Chassard , et al., “Original Salivary Sex Hormone Data of Naturally Menstruating Athletes and Hormonal Contraceptive Users,” BMJ Open Sport & Exercise Medicine 10, no. 4 (2024): e002078. 10.1136/bmjsem-2024-002078 PMC11575394 39564535

[20] A. Pelliccia , S. Sharma , S. Gati , et al., “2020 ESC Guidelines on Sports Cardiology and Exercise in Patients With Cardiovascular Disease,” European Heart Journal 42, no. 1 (2021): 17–96. 32860412 10.1093/eurheartj/ehaa605

[21] H. Persch and J. Steinacker , “Echocardiographic Criteria for Athlete's Heart With Cut‐Off Parameters and Special Emphasis on the Right Ventricle,” German Journal of Sports Medicine/Deutsche Zeitschrift fur Sportmedizin 71, no. 6 (2020): 151.

[22] A. L. Baggish , F. Wang , R. B. Weiner , et al., “Training‐Specific Changes in Cardiac Structure and Function: A Prospective and Longitudinal Assessment of Competitive Athletes,” Journal of Applied Physiology 104, no. 4 (2008): 1121–1128. 18096751 10.1152/japplphysiol.01170.2007

[23] R. De Bosscher , C. Dausin , K. Janssens , et al., “Rationale and Design of the PROspective Athletic Heart (Pro@Heart) Study: Long‐Term Assessment of the Determinants of Cardiac Remodelling and Its Clinical Consequences in Endurance Athletes,” BMJ Open Sport & Exercise Medicine 8, no. 1 (2022): e001309. 10.1136/bmjsem-2022-001309 PMC8935177 35368514

[24] J. C. van Hattum , S. M. Verwijs , S. M. Boekholdt , et al., “Elite: Rationale and Design of a Longitudinal Elite Athlete, Extreme Cardiovascular Phenotyping, Prospective Cohort Study,” BMJ Open Sport & Exercise Medicine 9, no. 1 (2023): e001505. 10.1136/bmjsem-2022-001505 PMC9900067 36756286

[25] N. Moulson , B. J. Petek , M. J. Ackerman , et al., “Rationale and Design of the ORCCA (Outcomes Registry for Cardiac Conditions in Athletes) Study,” Journal of the American Heart Association 12, no. 11 (2023): e029052. 37259981 10.1161/JAHA.122.029052 PMC10382007

[26] A. L. Spence , H. H. Carter , C. P. Murray , et al., “Magnetic Resonance Imaging‐Derived Right Ventricular Adaptations to Endurance Versus Resistance Training,” Medicine & Science in Sports & Exercise 45, no. 3 (2013): 534–541. 23073215 10.1249/MSS.0b013e3182780b0e

[27] F. D'Ascenzi , M. Solari , M. Biagi , et al., “P‐Wave Morphology Is Unaffected by Training‐Induced Biatrial Dilatation: A Prospective, Longitudinal Study in Healthy Athletes,” International Journal of Cardiovascular Imaging 32, no. 3 (2016): 407–415. 26474571 10.1007/s10554-015-0790-z

[28] A. Hodzic , P. Gendron , E. Baron , et al., “Inter‐Season Training Effects on Cardiovascular Health in American‐Style Football Players,” BMC Sports Science, Medicine and Rehabilitation 16, no. 1 (2024): 108. 10.1186/s13102-024-00888-4 PMC11092187 38741116

[29] F. D'Ascenzi , L. Cavigli , A. Marchese , et al., “Electrical and Structural Remodelling in Female Athlete's Heart: A Comparative Study in Women vs Men Athletes and Controls,” International Journal of Cardiology 400 (2024): 131808. 38262482 10.1016/j.ijcard.2024.131808

[30] A. Pelliccia , B. J. Maron , F. Culasso , et al., “Clinical Significance of Abnormal Electrocardiographic Patterns in Trained Athletes,” Circulation 102, no. 3 (2000): 278–284. 10899089 10.1161/01.cir.102.3.278

[31] M. Patrizio and G. Marano , “Gender Differences in Cardiac Hypertrophic Remodeling,” Annali dell′Istituto superiore di sanita 52, no. 2 (2016): 223–229. 10.4415/ANN_16_02_14 27364397

[32] S. N. Davis , “Effects of Gender on Neuroendocrine and Metabolic Counterregulatory Responses to Exercise in Normal Man,” Journal of Clinical Endocrinology & Metabolism 85, no. 1 (2000): 224–230. 10634391 10.1210/jcem.85.1.6328

[33] J. B. Carter , E. W. Banister , and A. P. Blaber , “The Effect of Age and Gender on Heart Rate Variability After Endurance Training,” Medicine & Science in Sports & Exercise 35, no. 8 (2003): 1333–1340. 12900687 10.1249/01.MSS.0000079046.01763.8F

[34] A. W. Bjerring , H. E. Landgraff , T. M. Stokke , et al., “The Developing Athlete's Heart: A Cohort Study in Young Athletes Transitioning Through Adolescence,” European Journal of Preventive Cardiology 26, no. 18 (2019): 2001–2008. 31284749 10.1177/2047487319862061

