# Supplementary material for: China Elite Athletes Cardiovascular hEath (China‐ACE) Study: A Protocol for a Multicenter Prospective Cohort
Source: Clin Cardiol. 2025 Dec 23;48(12):e70241. doi: 10.1002/clc.70241 (PMC12723624; doi:10.1002/clc.70241)
Supplement: Supplementary file 1 — ptorocol supplemtary material. [file CLC-48-e70241-s001.docx]

**China elite Athletes Cardiovascular hEath (China-ACE) study: A protocol for a multi-center prospective cohort**

Sheng Xu^1#^, Yaodong Guo^2#^, Hao Xin^1^, Shanshan Zhuo^1^, Yang Zhao^1^, Yanna Jiang^1^, Yuanqi Huang^2^, Chunyan Huang^3^, Tao Chen^4^, Qi Chen^4^, Qianru Zhao^5^, Dan Wang^2^, Xiao Liu^6,7*^, Zhiwei Yan^2,4*^

1: Department of Ultrasound Medicine, Jinqiu Hospital, Shenyang, Liaoning, China.

2: School of Physical Education and Sport Sciences, Fujian Normal University, Fuzhou, Fujian, China.

3: Department of Ultrasound, the First Affiliated Hospital of Fujian Medical University, Fuzhou, Fujian, China

4: Liaoning Provincial Sports Development Center, Shenyang, Liaoning, China

5: Shenyang Sports Research and Medical Center, Shenyang Sports Development Center, Shenyang, Liaoning, China

6: Department of Cardiology, Sun Yat-sen Memorial Hospital of Sun Yat-sen University, Guangzhou, China

7: Cardiovascular and Metabolic Disorders Program, Duke-National University of Singapore Medical School, Singapore, Singapore

^#^ Contributed equally

^*^Correspondence:

Xiao Liu, Department of Cardiology, Sun Yat-sen Memorial Hospital of Sun Yat-sen University, Guangzhou 510000, China; Cardiovascular and Metabolic Disorders Program, Duke-National University of Singapore Medical School, Singapore, Singapore. Email: kellyclarkwei@vip.qq.com

Zhiwei Yan, School of Physical Education and Sport Sciences, Fujian Normal University, Fuzhou, Fujian, China; Liaoning Provincial Sports Development Center, Shenyang, Liaoning, China. Email: [zhiweiyan163@163.com](mailto:zhiweiyan163@163.com).

**SUPPLEMENTAL METHODS**

**Training Load Assessment**

To describe the relationship between exercise dose and cardiovascular adaptation accurately, this study will employ quantitative and semi-quantitative methods to monitor exercise load over one year. Given the diversity of sports in this study, the exercise load exhibits sport-specific variations, making it impractical to apply a single standardized measurement method to all participants. Therefore, this study will implement a comprehensive exercise load assessment framework that integrates sport-specific quantification methods tailored to different sports. Specifically, the exercise load will be evaluated via heart rate (HR) -based metrics, resistance load monitoring, and the rating of perceived exertion scale (RPE), ensuring a thorough and accurate assessment of the training intensity and volume.

Athletes will be provided with a numbered HR monitor (Polar Electro, Kempele, Finland) for retention during the investigation period. The athletes are instructed to wear it during endurance and metabolic training sessions for the purpose of logging HR and session durations. The researcher then downloaded the HR data files on a weekly basis, which were cross-referenced with diary contents to ensure accurate data registration.

Strength training is required for any current sport. Therefore, this study will also document the resistance load of athletes during the research period. Training data—including training content, repetitions, sets, resistance load, and rest intervals—will be collected through training logs. Given the variability in training levels among individuals, resistance load will be further standardized on the basis of the 1RM of athletes. The absolute training volume and intensity relative training volume for each resistance training session will be calculated using the following formulas(1).

$$\text{Absolute Training Volume}\text{=Load}\text{ }\text{×}\text{ }\text{Repetitions}\text{ }\text{×}\text{ }\text{Sets}$$

$$\text{Intensity Relative Training Volume }\text{=}\text{Load}_{\text{\%1RM}}\text{ }\text{×}\text{ }\text{Repetitions}\text{ }\text{×}\text{ }\text{Sets}$$

Previous studies have demonstrated the validity and reliability of sRPE in quantifying exercise load across various sports, including basketball, swimming, beach volleyball, wrestling, judo, canoeing, cycling, boxing, and rowing(2-10). In this study, participants will be required to complete the Borg-10 ratings of the RPE scale, designed by Foster, within 30 min after each training session or competition to assess their perceived exertion. Additionally, the total training duration, from the beginning to the end of the session, will be recorded. The sRPE will then be calculated using the following formula.

$\text{sRPE}\text{ }\text{=}\text{ }\text{duration}\text{ }\text{×}\text{ }\text{RPE}$

The training duration is measured in min, and the RPE is an arbitrary unit (AU).

**Body composition**

The body composition of athletes will be tested using bioelectrical impedance analysis by the body composition analyzer (InBody 770, InBody Co, Korea), which has been validated for its effectiveness in body composition assessment(11). The specific measurement procedure follows our previous study(12). The participant will stand barefoot on the platform for the initial weight measurement. The participant will then hold the handle of the device with their thumb and fingers to ensure complete contact with the electrodes, with the arms naturally turned away from the body. The participant will be asked to stand still for approximately one minute throughout the measurement. The main outcomes will include total fat mass, total muscle mass, and body fat percentage.

**Cardiovascular function**

The cardiovascular function of athletes will be analyzed by blood pressure, rate-pressure product, and brachial-ankle pulse wave velocity (baPWV) using the automated sphygmomanometer (Omron HEM 3, Omron, Japan) and vascular function analyzer (BP-203RPEⅢ, Omron, Japan). Athletes will be asked to avoid strenuous physical activity and take diets that affect cardiovascular reactivity (such as high-nitrate foods, alcohol, and coffee). To avoid the impact of training on measurement, athletes will be tested on rest days. Athletes will remain in a natural seated position for at least 10 min before the measurement. Blood pressure will be measured three times at the brachial artery of the left upper arm, and the average of the last two readings will be taken as the final result. During the measurement, the pneumatic cuff will be placed on both ankles and the humerus of the athletes, the electrocardiograph electrodes will be placed on both wrists. The distance between two points will be automatically estimated based on the height of the athlete, while the time difference between the beginning of the rise point of the brachial and tibial pulse waveform during contraction will be measured. The baPWV will be defined as the distance divided by the time difference. High baPWV is indicative of atherosclerosis, and we will record high baPWV on both sides for analysis.

**ECG**

The cardiovascular function of athletes will be tested using the 12-lead ECG (EDAN SE-1010, China). To avoid the impact of training on measurement, athletes will be tested on rest days. In addition, athletes will be asked to avoid alcohol, caffeine, and tobacco for at least 24 hours before the measurement. Before the ECG measurements are taken, athletes will be required to rest in a quiet room for at least 20 min to minimize the influence of physical activity on the ECG results. During the measurement, athletes will lie in a supine position and standard 12-lead connection methods will be used to attach the limb and chest leads. All leads will be expected to produce clear ECG signals without muscle tremor artifacts or electromagnetic interference, and the electrodes will need to maintain good contact. The ECG data of athletes will be classified and processed based on the international standards(13). The following ECG variables will be measured and recorded, and the corresponding parameters will be calculated: HR, P-wave duration (ms), P-wave amplitude (mV), PQ duration (ms), R-wave axis, QRS duration (ms), QTc duration (ms), RV5+SV1 (mV), and the QTc will be corrected using Bazett's formula(14).

$$\text{QTc=QT÷√(R-R)}$$

**Cardiac autonomic function**

The cardiac autonomic nervous function of athletes will be tested using HRV by the 12-lead ECG (EDAN SE-1010, China), which has been validated for its effectiveness in cardiac autonomic function(15). To avoid the impact of training on measurement, athletes will be tested on rest days. In addition, athletes will be asked to avoid alcohol, caffeine, and tobacco for at least 24 hours before the measurement. Throughout the measurement, athletes will be asked to take a comfortable sitting position, remain awake, and avoid physical activity. They will be allowed to breathe freely without speaking, as normal spontaneous breathing is unlikely to cause significant differences in heart rate-derived metrics compared to controlled breathing(16). HRV will be measured during the last 5 min of 10 min recording period to ensure stable data collection. HRV analysis will include time-domain, frequency-domain, and nonlinear measures(17). Time-domain indices include: SDNN, RMSSD, RR_max_, RR_mean_, RR_min_, Tempo (s), Misure, NN50, PNN50, and SMisure. Frequency-domain analysis was conducted using the fast Fourier transform (FFT) based on Welch’s periodogram method (window width: 300 s; window overlap: 50%; frequency domain points: 300 points/Hz). The R-R intervals were analyzed as VLF: 0–0.04 Hz, LF: 0.04–0.15 Hz, and HF: 0.15–0.40 Hz components. Frequency domain parameters were expressed in absolute values (ms^2^), normalized to total power (nu), and logarithmically transformed absolute values (log ms^2^). Non-linear indicator analysis includes: SD1, SD2, SD1/SD2, and STD. For female athletes, measurements will be scheduled during the follicular phase to standardize hormonal status and minimize the influence of within-cycle estrogen fluctuations. Meantime, given the effects of hormonal contraceptives on the autonomic nervous system, we will exclude female participants who used hormonal contraceptives during the one-year follow-up period.

**Echocardiography**

All echocardiographic examinations are performed on a GE VIVID E95 ultrasound system (GE Vingmed Ultrasound, Horten, Norway), equipped with a Probe M3S (1.0-4.0 MHz). The athlete adopts a left lateral position with a synchronized ECG connection for conventional echocardiographic examination. Two-dimensional images, including parasternal long-axis view, apical four-chamber view, apical two-chamber view, and aortic short-axis view images, were acquired for measurement. The interventricular septal thickness (IVST), left ventricular posterior wall thickness (LVPWT), left ventricular end-diastolic diameter (LVEDD), left ventricular end-systolic diameter, left atrial diameter, aortic root diameter, and ascending aorta diameter are measured in the parasternal long-axis view image. The pulmonary artery diameter is measured in the short-axis view of the aorta. The right atrium superior-inferior diameter, right atrium transverse diameter, right ventricle basal diameter, and right ventricle mid-cavity diameter are measured at the apical four-chamber view image. The left ventricular end-diastolic volume (LVEDV) and left ventricular end-systolic volume (LVESV) are measured using the biplane Simpson's method in the apical four-chamber view and the apical two-chamber view images. The left ventricular ejection fraction is calculated using the following formula:

$$\text{left ventricular ejection fraction=(LVDEV-LVESV)/LVEDV}$$

Relative wall thickness is calculated as.

$$\text{R}\text{elative wall thickness}\text{ = 2 ×}\text{ }\text{LVPWT}\text{ }\text{/}\text{ }\text{LVEDD}$$

Left ventricular mass is calculated by the following formula.

$$\text{L}\text{eft ventricular mass}\text{ =}\text{ }\text{0.8}\text{ }\text{× 1.04 × [(IVST + LVEDD + LVPWT)}\text{3}\text{ - LVEDD}\text{3}\text{] + 0.6g}$$

Left ventricular mass index was calculated as the ratio of left ventricular mass to BSA.

In pulsed Doppler imaging mode, measurements are taken at the apical four-chamber, apical five-chamber, and aortic short-axis views to measure the peak E wave velocity, peak A wave velocity, aortic valve flow peak velocity, and pulmonary artery valve flow peak velocity, and the E/A ratio is calculated. In tissue Doppler imaging mode, the early diastolic mitral annular velocity and late diastolic mitral annular velocity are measured at the lateral and septal mitral annulus from the apical four-chamber view, and the average velocity of the mitral annulus and the E/e' ratio are calculated. Tricuspid annular plane systolic excursion is measured at the apical four-chamber view by M-mode.

**Circulatory biochemical measurements**

During each laboratory visit, athletes were instructed to arrive at the laboratory at 8:00 a.m. in a fasted state. A trained phlebotomist collected a 2 mL venous blood sample from the antecubital vein into a serum separation tube. The blood samples were allowed to clot at room temperature for 30 min and then centrifuged at 2200 × g for 10 min at 4^°^C. The resulting serum was aliquoted and stored at −80^°^C until batch analysis. Importantly, blood sampling for female athletes will be scheduled during the follicular phase.

The white blood cell count, red blood cell count, hemoglobin concentration, hematocrit, lymphocyte count, monocyte count, granulocyte count, and platelet count will be measured using a fully automated hematology analyzer (MEK-6410P, Nihon Kohden, Japan). Aspartate aminotransferase, amylase, lipase, lactate dehydrogenase, creatine kinase, creatine kinase-MB, α-hydroxybutyrate dehydrogenase, high-density lipoprotein, low-density lipoprotein, triglyceride, total cholesterol, uric acid, urea, urea/creatinine, glucose, total CO_2_, calcium, phosphorus, Mg, K^+^, Na^+^, Cl^-^, and C-reactive protein will be detected using a fully automated biochemical analyzer (SD1, Seamaty, China) via microfluidic method.

**Sex hormones**

The procedure for testing sex hormone markers was consistent with that described in Circulation Markers in terms of blood collection methods and sample handling. The levels of testosterone, progesterone, sex hormone-binding globulin, follicle-stimulating hormone, estradiol, and luteinizing hormone in athletes will be tested using corresponding test kits, with males tested for testosterone only. All kits will be purchased from Jiangsu Enzyme Exemption Industry CO., Ltd. (testosterone: MM-0779H1, progesterone: MM-0774H1, sex hormone-binding globulin: MM-0764H1, follicle-stimulating hormone: MM-1015H1, estradiol: MM-0777H1, luteinizing hormone: MM-0793H1). In addition, given the impact of hormonal contraceptives on sex hormone levels, we will exclude female participants who used hormonal contraceptives during the one-year follow-up period.

**Cardiopulmonary exercise test (CPET)**

To accurately test the cardiorespiratory fitness levels of different athletes and their relationship with cardiovascular adaptation, this study will measure VO_2max_ through CPET. During test preparation, athletes should rest in a seated or supine position for 5–10 min to measure and record HR, blood pressure, resting VO₂, carbon dioxide output, minute ventilation, and baseline pulmonary function parameters as baseline data. After completing the resting measurements, athletes will perform an 8-10 min self-paced warm-up on the designated testing equipment.

Notably, weightlifting, wrestling, and judo rely less on VO_2max_, so strength-based athletes are not included in the VO_2max_ measurements. Additionally, due to the significant differences in physiological and biomechanical characteristics among different sports, a uniform VO_2max_ measurement method cannot fully reflect the specificity of each sport. Rowing and canoeing share similar movement patterns and physiological characteristics with rowing ergometry, whereas the cycling phases of triathlon and competitive cycling resemble power cycling ergometry. Moreover, sports such as swimming, basketball, badminton, and beach volleyball involve full-body movement patterns. Based on these considerations, this study will use rowing ergometry, a cycle ergometer, and treadmill testing for measurements.

The CPET tests on the cycle ergometer and treadmill will follow the Bruce exercise test protocol. The initial power output, power increment per minute, treadmill incline, and pedaling cadence will be determined based on the specific sport. The test will continue until the respiratory exchange ratio exceeds the preset thresholds (1.1 for basketball, beach volleyball, and triathlon thresholds, 1.15 for cycling thresholds, and 1.0 for swimming thresholds) and the athlete will reach voluntary exhaustion(18-23). Throughout the test, a gas analyzer (VO2000, MedGraphics Corp, USA) will be used to perform breath-by-breath respiratory gas exchange analysis. VO_2max_ will be determined as the highest oxygen uptake value recorded during the last 30 seconds of the test.

The CPET tests on the rowing ergometer will use an intermittent incremental load protocol(24), where each stage consists of 2 min of rowing followed by 30 seconds of rest. Considering sex differences in strength, the initial power output will be set at 200 W for males and 150 W for females, with an increment of 50 W per stage. Athletes must reach the target power within the first 15 s and maintain it for the remaining 1 min and 45 s until the respiratory exchange ratio exceeds 1.15, reaching voluntary exhaustion. Exhaled gases will be collected and analyzed by a gas analyzer to determine metabolic and ventilatory parameters.

The criteria for determining voluntary exhaustion in the above exercise tests include the following: VO₂ reaches a plateau despite the continuous increase in exercise load. The participant is unable to continue despite verbal encouragement.

**Skeletal muscle function**

The bilateral quadriceps muscle function of athletes will be tested using the Myoton PRO (Myoton AS, Tallinn, Estonia), which has been validated for its effectiveness in skeletal muscle function(25). The Myoton PRO probe will be gently placed on the bulge, ensuring that it is perpendicular to the muscle surface. Constant pressure will be applied to trigger slight muscle oscillations, and the oscillations detected under the probe allow for the calculation of tissue viscoelastic properties, as well as muscle tension, elasticity, and stiffness.

**Physical Performance：**

Considering the specificity of physical performance assessment methods across different sports, this study will select the most appropriate assessment methods for each sport. The weightlifting will allow athletes to self-select the lifts for performance testing, including the snatch, clean and jerk, and deadlift. Coaches will record the completion time and the maximum weight lifted(26). For wrestling, the measurement will be conducted using a dummy throw test, lasting for 3 min. This test alternates between a slow mode (4 compulsory suplex throws in 30 s) and fast mode (performing as many suplex throws as possible in 15 s). Coaches will record the total number of valid throws in the fast mode(27). The judo event will be assessed using the Special Judo Fitness Test, consisting of three consecutive rounds: the first round lasts 15 seconds, whereas the next two rounds last 30 seconds each, with a 10-second rest between rounds. During the test, the athlete moves quickly between two fixed partners of similar body weight (positioned 3 meters apart) and performs as many Ippon Seoinage throws as possible. Finally, two coaches will record the total number of valid throws, and performance will be evaluated using the following formula(28).

$$\text{I}\text{ndex=}\frac{\text{final }\text{HR }\left( \text{bpm} \right)\text{+}\text{HR}\text{ 1}\min\text{after SJFT (bpm)}}{\text{number of throws}}$$

Rowing (2000 m) and canoeing (1000 m) will be evaluated through a rowing ergometer time trial. At the end of the race, the following metrics will be recorded: total time, average power output, average stroke rate, average pace per 500 meters, and distance per stroke(29). Swimming will be assessed through a 200-meter freestyle time trial. All tests will be conducted in the same swimming pool, with a diving start from the starting block. Coaches will record the completion time for evaluation(30). Cycling will be evaluated using a bicycle dynamometer for a 20 min trial. During this time, the athlete will be able to check the pedaling frequency, gear settings, and elapsed time, and upon completion of the test, the power output and distance ridden will be recorded(31). The triathlon will be conducted as a simulated race, with the completion time recorded(32).

Vertical jumps and 5-10-5 agility drills are used as a way to assess physical performance in beach volleyball(33). Both tests will be conducted on the beach. The vertical jump test is divided into 3 sessions, each 1 minute apart, with the vertical jump height measured and the best score recorded by the coach. The 5-10-5 yard run consists of three cones placed 5 yards apart in a straight line. The athlete starts at the center cone, sprints to either end cone, changes direction to return to the center, sprints to the other end cone, and finally returns to the center cone. Athletes will perform 2 trials, 2 minutes apart, and will have their completion time recorded by the coach. Basketball will use timed 282 feet sprint and shooting accuracy to reflect physical performance. The timed 282-foot sprint is determined by a 282-foot round trip run (baseline-half-court-baseline-full-court-baseline) with the coach recording the completion time. The shooting accuracy is determined by free throws (15 feet) and 15 three-point shots(34). Badminton will use the low center of gravity running rope and double swing jump rope to assess physical performance(35). The low center of gravity running test consists of the athlete starting from the center of the court and running to each of the 4 corners (right front court, left backcourt, left front court, right backcourt) in turn. Each time the athlete returns to the center, the athlete is required to touch the singles sideline and then immediately turn to the next target, terminating at the end of 20 repetitions. Double pendulum rope skipping requires athletes to swing the rope with both hands and pass the rope through their feet twice per jump. If the rope skipping is interrupted for subjective reasons, the counter will be stopped until 1500 jumps are completed, and the final record of completion time will be obtained. Given the validated effectiveness of accelerometers in boxing physical performance, we fixed accelerometers to the inside wrist of boxing gloves to assess the maximal striking velocity of the athletes(36). Each boxer completed different types of punches (e.g., straight punches) three times each at maximum velocity, and data were collected for subsequent analysis.

**References**

1. Scott BR, Duthie GM, Thornton HR, Dascombe BJ. Training monitoring for resistance exercise: theory and applications. Sports medicine. 2016;46:687-98.

2. Sansone P, Tschan H, Foster C, Tessitore A. Monitoring Training Load and Perceived Recovery in Female Basketball: Implications for Training Design. J Strength Cond Res. 2020;34(10):2929-36.

3. Borges TO, Bullock N, Duff C, Coutts AJ. Methods for quantifying training in sprint kayak. J Strength Cond Res. 2014;28(2):474-82.

4. Sanders D, Abt G, Hesselink MKC, Myers T, Akubat I. Methods of Monitoring Training Load and Their Relationships to Changes in Fitness and Performance in Competitive Road Cyclists. Int J Sports Physiol Perform. 2017;12(5):668-75.

5. DellaValle DM, Haas JD. Quantification of training load and intensity in female collegiate rowers: validation of a daily assessment tool. J Strength Cond Res. 2013;27(2):540-8.

6. Nobari H, Badicu G, Akyildiz Z, Clemente FM. Relationships between training load and wellbeing measures across a full season: a study of Turkish national youth wrestlers. Biol Sport. 2023;40(2):399-408.

7. Agostinho MF, Philippe AG, Marcolino GS, Pereira ER, Busso T, Candau RB, Franchini E. Perceived training intensity and performance changes quantification in judo. J Strength Cond Res. 2015;29(6):1570-7.

8. Tometz MJ, Jevas SA, Esposito PM, Annaccone AR. Validation of Internal and External Load Metrics in NCAA D1 Women's Beach Volleyball. J Strength Cond Res. 2022;36(8):2223-9.

9. Surała O, Malczewska-Lenczowska J, Sitkowski D, Witek K, Słomiński P, Certa M, Madej D. Effect of training load on sleep parameters and biochemical fatigue markers in elite swimmers. Biol Sport. 2023;40(4):1229-37.

10. Uchida MC, Teixeira LF, Godoi VJ, Marchetti PH, Conte M, Coutts AJ, Bacurau RF. Does the Timing of Measurement Alter Session-RPE in Boxers? J Sports Sci Med. 2014;13(1):59-65.

11. Czuba M, Wilk R, Karpiński J, Chalimoniuk M, Zajac A, Langfort J. Intermittent hypoxic training improves anaerobic performance in competitive swimmers when implemented into a direct competition mesocycle. PLoS One. 2017;12(8):e0180380.

12. Yu P, Zhu Z, He J, Gao B, Chen Q, Wu Y, et al. Effects of high-intensity interval training, moderate-intensity continuous training, and guideline-based physical activity on cardiovascular metabolic markers, cognitive and motor function in elderly sedentary patients with type 2 diabetes (HIIT-DM): a protocol for a randomized controlled trial. Front Aging Neurosci. 2023;15:1211990.

13. Drezner JA, Sharma S, Baggish A, Papadakis M, Wilson MG, Prutkin JM, et al. International criteria for electrocardiographic interpretation in athletes: Consensus statement. Br J Sports Med. 2017;51(9):704-31.

14. Bazett H. An analysis of the time‐relations of electrocardiograms. Annals of noninvasive electrocardiology. 1997;2(2):177-94.

15. Stein P, PK, Kleiger M, RE. Insights from the study of heart rate variability. Annual review of medicine. 1999;50(1):249-61.

16. Shaffer F, Ginsberg JP. An overview of heart rate variability metrics and norms. Frontiers in public health. 2017;5:258.

17. Pham T, Lau ZJ, Chen SA, Makowski D. Heart rate variability in psychology: A review of HRV indices and an analysis tutorial. Sensors. 2021;21(12):3998.

18. Charitonidis K, Koutlianos N, Anagnostaras K, Anifanti M, Kouidi E, Deligiannis A. Combination of novel and traditional cardiorespiratory indices for the evaluation of adolescent volleyball players. Hippokratia. 2019;23(2):70-4.

19. Sunderland KL, Greer F, Morales J. VO2max and ventilatory threshold of trained cyclists are not affected by 28-day L-arginine supplementation. J Strength Cond Res. 2011;25(3):833-7.

20. Galy O, Manetta J, Coste O, Maimoun L, Chamari K, Hue O. Maximal oxygen uptake and power of lower limbs during a competitive season in triathletes. Scand J Med Sci Sports. 2003;13(3):185-93.

21. Wu S, Jiang H. Examining the impact of differing caffeine dosages in conjunction with plyometric training on physiological adaptations in basketball players. Sci Rep. 2024;14(1):15571.

22. Rodríguez FA, Truijens MJ, Townsend NE, Stray-Gundersen J, Gore CJ, Levine BD. Performance of runners and swimmers after four weeks of intermittent hypobaric hypoxic exposure plus sea level training. J Appl Physiol (1985). 2007;103(5):1523-35.

23. Pelarigo JG, Fernandes RJ, Ribeiro J, Denadai BS, Greco CC, Vilas-Boas JP. Comparison of Different Methods for the Swimming Aerobic Capacity Evaluation. J Strength Cond Res. 2018;32(12):3542-51.

24. Huntsman HD, DiPietro L, Drury DG, Miller TA. Development of a rowing-specific VO2max field test. J Strength Cond Res. 2011;25(6):1774-9.

25. Gacto-Sánchez M, Medina-Mirapeix F, Benítez-Martínez JC, Montilla-Herrador J, Palanca A, Martín-San Agustín R. Estimating quadriceps and hamstrings strength through myoton among recreational athletes. Journal of Sport Rehabilitation. 2023;32(7):827-33.

26. Greene DA, Varley BJ, Hartwig TB, Chapman P, Rigney M. A Low-Carbohydrate Ketogenic Diet Reduces Body Mass Without Compromising Performance in Powerlifting and Olympic Weightlifting Athletes. J Strength Cond Res. 2018;32(12):3373-82.

27. Durkalec-Michalski K, Zawieja EE, Podgórski T, Zawieja BE, Michałowska P, Łoniewski I, Jeszka J. The Effect of a New Sodium Bicarbonate Loading Regimen on Anaerobic Capacity and Wrestling Performance. Nutrients. 2018;10(6).

28. Filip-Stachnik A, Krawczyk R, Krzysztofik M, Rzeszutko-Belzowska A, Dornowski M, Zajac A, et al. Effects of acute ingestion of caffeinated chewing gum on performance in elite judo athletes. J Int Soc Sports Nutr. 2021;18(1):49.

29. Garnacho-Castaño MV, Pleguezuelos-Cobo E, Berbel M, Irurtia A, Carrasco-Marginet M, Castizo-Olier J, et al. Effects of acute beetroot juice intake on performance, maximal oxygen uptake, and ventilatory efficiency in well-trained master rowers: a randomized, double-blinded crossover study. J Int Soc Sports Nutr. 2024;21(1):2373170.

30. Wilson EE, McKeever TM, Lobb C, Sherriff T, Gupta L, Hearson G, et al. Respiratory muscle specific warm-up and elite swimming performance. Br J Sports Med. 2014;48(9):789-91.

31. MacInnis MJ, Thomas ACQ, Phillips SM. The Reliability of 4-Minute and 20-Minute Time Trials and Their Relationships to Functional Threshold Power in Trained Cyclists. Int J Sports Physiol Perform. 2019;14(1):38-45.

32. Luckin-Baldwin KM, Badenhorst CE, Cripps AJ, Landers GJ, Merrells RJ, Bulsara MK, Hoyne GF. Strength Training Improves Exercise Economy in Triathletes During a Simulated Triathlon. Int J Sports Physiol Perform. 2021;16(5):663-73.

33. Holtgeerts RN, Gann J, Jung HC, Hey W. The Impact of Recovery Time on Performance in Division I Collegiate Beach Volleyball Players. J Strength Cond Res. 2022;36(3):667-73.

34. Mah CD, Mah KE, Kezirian EJ, Dement WC. The effects of sleep extension on the athletic performance of collegiate basketball players. Sleep. 2011;34(7):943-50.

35. Zhao W, Wang C, Bi Y, Chen L. Effect of Integrative Neuromuscular Training for Injury Prevention and Sports Performance of Female Badminton Players. Biomed Res Int. 2021;2021:5555853.

36. López-Laval I, Sitko S, Muñiz-Pardos B, Cirer-Sastre R, Calleja-González J. Relationship Between Bench Press Strength and Punch Performance in Male Professional Boxers. J Strength Cond Res. 2020;34(2):308-12.
